# Prediction of air quality index based on the SSA-BiLSTM-LightGBM model

**DOI:** 10.1038/s41598-023-32775-2

**Published:** 2023-04-05

**Authors:** Xiaowen Zhang, Xuchu Jiang, Ying Li

**Affiliations:** Zhongnan University of Economics and Law, Wuhan, 430073 China

**Keywords:** Climate sciences, Environmental sciences, Environmental social sciences, Engineering, Mathematics and computing

## Abstract

The air quality index (AQI), as an indicator to describe the degree of air pollution and its impact on health, plays an important role in improving the quality of the atmospheric environment. Accurate prediction of the AQI can effectively serve people’s lives, reduce pollution control costs and improve the quality of the environment. In this paper, we constructed a combined prediction model based on real hourly AQI data in Beijing. First, we used singular spectrum analysis (SSA) to decompose the AQI data into different sequences, such as trend, oscillation component and noise. Then, bidirectional long short-term memory (BiLSTM) was introduced to predict the decomposed AQI data, and a light gradient boosting machine (LightGBM) was used to integrate the predicted results. The experimental results show that the prediction effect of SSA-BiLSTM-LightGBM for the AQI data set is good on the test set. The root mean squared error (RMSE) reaches 0.6897, the mean absolute error (MAE) reaches 0.4718, the symmetric mean absolute percentage error (SMAPE) reaches 1.2712%, and the adjusted R^2^ reaches 0.9995.

## Introduction

The phenomenon of air pollution seriously affects human health and life safety. The World Health Organization and the United Nations have called on all countries to work to reduce air pollution and improve air quality. Accurate air quality prediction can effectively serve people's lives, reduce pollution control costs and become an important breakthrough to improve the quality of the environment. Therefore, realizing the high-precision prediction of the air quality index (AQI), which has positive significance for urban development and national health, is an important research topic.

In recent years, many scholars at home and abroad have conducted in-depth research on AQI prediction. First, the main methods used are numerical methods and traditional statistical models. The core of the numerical method is based on atmospheric dynamics and environmental chemistry, and models are constructed and solved by computers based on air pollution emission source data and meteorological data. However, the modeling and calculation process of this method is complicated, and it is difficult to popularize in practical applications, so it is not discussed in this paper. The core of traditional statistical models is to predict based on historical data, and the modeling process is relatively convenient and mature, so it is favored by scholars at home and abroad. However, with the improvement of artificial intelligence algorithms, many scholars at home and abroad have proposed a series of machine learning prediction methods based on studying the above two methods. It is now divided into three main categories according to different predictive models:

The first category uses traditional statistical models for prediction. He R R et al.^1^ established the autoregressive integrated moving average model (ARIMA) for time series prediction and obtained residual series for further analysis. Sigamani S et al.^2^ established a multiple linear regression model to predict AQI based on the correlation between air pollutants and two time-series related variables of meteorological parameters. Jiao D F et al.^3^ used the exponential regression analysis model to predict the AQI after model selection by Box‒Cox transformation of each variable. Yang X et al.^4^ proposed a long-term prediction model for Beijing haze by time series analysis. By constructing the dynamic structural econometric model of the daily increment of haze, it was simplified into a vector autoregressive model. The research results show that the model performs very well in the AQI prediction of the next day. However, because the AQI is affected by many difficult-to-decide and nonlinear factors, the efficiency and accuracy of traditional regression prediction methods may be poor in this type of prediction.

The second category is the machine learning framework for prediction. Zhang C et al.^5^ proposed a recursive neural network model based on a long short-term memory unit to accurately predict the AQI of air quality by effectively utilizing the ability of long-distance dependence information in time series data and combining it with pollution index factors. Hua H D et al.^6^ introduced a Bayesian network into the study of air quality and predicted the AQI through a Bayesian network. Kumar A et al.^7^ used neural networks based on Principal component analysis (PCA) to predict daily AQI. The AQI of standard air pollutants was predicted using the AQI of the previous day and meteorological variables. Ganesh S S et al.^8^ used a radial basis function model trained by conjugate gradient descent to predict the AQI of a specific region of interest and found that this model outperformed other artificial neural networks (ANNs).

The third category is the combined prediction model based on the above models: Zhao X et al.^9^ predicted the AQI in a nonmonitored area from the temporal and spatial dimensions. The improved K-nearest neighbor (KNN) algorithm is applied to predict the AQI value between monitoring stations in the temporal dimension model. Meanwhile, the backpropagation neural network considering geographical distance is introduced to predict the AQI in the spatial dimension. Xu T et al.^10^ first evaluated and analyzed the impact of major pollutants on air quality through grey correlation analysis.Then, an improved seagull optimization algorithm was proposed and combined with support vector regression, and the improved seagull optimization algorithm (ISOA) was proposed and combined with support vector regression (SVR) to establish a hybrid predicted model ISOA-SVR. Zhu J et al.^11^ proposed a new hybrid multi-point prediction method by combining K-means clustering with recurrent neural network and long short-term memory (RNN-LSTM) model. Compared with traditional methods, this method improved the accuracy and effectiveness of prediction and revealed the relationship between land use and air quality index. Chhikara P et al.^12^ predicted the AQI through a distributed federated learning (FL) algorithm. Liu X et al.^13^ established the LSTM-SSA model to improve the prediction accuracy through long short-term memory(LSTM) coupled with sparrow search algorithm (SSA). Later, it was found that the AQI data was not stable. To enhance the prediction effect based on this characteristic, many scholars have proposed different schemes. For example, Yan K et al.^14^ proposed the CEEMD-WOA-Elman model. By using complementary ensemble empirical mode decomposition (CEEMD) to decompose the nonstationary AQI sequence into multiple stationary eigenmode function components and then using the whale optimization algorithm (WOA)-optimized Elman neural network for prediction, the effect of nonstationarity can be effectively reduced, and accurate prediction of the air quality level can be achieved. Wang Z et al.^15^ proposed an interval-valued AQI prediction model based on double decomposition and the optimal combination-ensemble learning method. Ji C et al.^16^ decomposed and reconstructed AQI sequences using complete ensemble empirical mode decomposition adaptive noise (CEEMDAN) and sample entropy (SE). Then, according to the characteristics of high and low frequencies, they used LSTM to predict high-frequency components and a regularized extreme learning machine (RELM) to predict low-frequency terms. Furthermore, the modified WOA was used to optimize the hyperparameters of the model. Dai Hongbin et al.^17^ proposed an XGBoost-GARCH-MLP mixed model combining XGBoost, GARCH models and MLP models to predict PM2.5 concentration and volatility. The proposed model has good performance in the long-term forecasting process. Dai Hongbin et al.^18^ used the matter-element extension (MEE) model to evaluate the haze hazard risk levels in different cities, and the indicator weights were determined by improving the principal component analysis (PCA) method using the entropy weight method. Finally, several risk assessment models were established by improving the particle swarm optimization (IPSO) light gradient boosting machine (LightGBM) algorithm.

In summary, by combining the relevant literature on AQI prediction, it can be found that ① Compared with traditional statistical models, machine learning and deep learning models can deal with time series prediction more accurately. However, due to the nonstationary nature of AQI data, it may be difficult for a single model to fully explore the internal regularity between data. ② In view of the nonstationary nature of AQI data, most of the existing studies use EMD, CEEMD and CEEMDAN to decompose it. ③ After decomposition, most of the current studies realize the integration of prediction results by directly adding decomposed forecast data, and there is a lack of new methods for the integration of forecast results after decomposition.

Aiming at the instability of AQI data, this paper proposes a hybrid prediction model that combines SSA decomposition, BiLSTM prediction and LightGBM integration. First, SSA is a method to process nonlinear time series data. Based on the singular value decomposition (SVD) of a specific matrix, the trend, oscillation component and noise can be decomposed to reduce the influence of data instability on the prediction effect. BiLSTM adds a reverse operation based on LSTM, which is better than LSTM at capturing the relationship between sequential features. However, for time series prediction, it may be decided jointly by the previous input and the later input to make the prediction more accurate. Finally, the idea of stacking is introduced in this paper, and the LightGBM model is used to integrate the forecast data. The experimental results show that this model has a good prediction effect.

In this paper, AQI data were predicted. The objective of this study is to provide a scientific, effective, accurate and robust AQI prediction method that is intended to provide data support for the decision-making of meteorological departments and relevant ecological environmental management departments. Meteorological departments and relevant ecological environment management need to grasp the spatial distribution and accumulation of air pollutants as well as the seasonal changes over time. Accurate AQI prediction enables relevant administrative departments to take precautions against possible air pollution in advance and make targeted deployments to relevant enterprises, standardize pollution emission policies, and formulate and publish reasonable and scientific air treatment policies and plans. At the same time, the accurate prediction of AQI in the meteorological department can also put forward some guiding opinions for residents' life planning to minimize the damage to residents’ respiratory system and cardiovascular system caused by the deterioration of air quality.

The main contributions of this paper to the literature are as follows: ① As far as we know, most existing studies have used EMD or various optimization decomposition models based on EMD to decompose unstable AQI data. However, through model comparison, it is found that SSA decomposition has a better prediction effect than the former. Therefore, this paper introduces SSA into the prediction study of AQI to capture series features more effectively. ② Most of the previous studies on AQI forecasting directly add decomposed forecast data to obtain forecast results, and there is a lack of new methods for the integration of decomposed forecast results. This paper refers to the idea of stacking and introduces the LightGBM model to integrate the data. The experimental results show that the prediction effect of this method is significantly increased compared with the previous direct addition model. ③ A new AQI prediction mixed model based on SSA-BiLSTM-LightGBM is constructed in this paper. Compared with the previous model, the prediction effect is significantly increased.

## Models

### SSA

SSA^19^ is a method to deal with nonlinear time series data. SSA decomposes the trend, oscillation component and noise from a time series based on the SVD of a specific matrix constructed on the time series. For a time series of length *N*. $X=({x}_{1},...,{x}_{n})$*, N* > *2*, and *X* is a nonzero series. Let the integer *L (1* < *L* < *N)* be the window length and *K* = ${N}_{L}$+ *1*. The whole process of the SSA algorithm consists of two complementary stages: decomposition and reconstruction. The basic process is as follows:

#### Embed

The original time series is mapped to a sequence of vectors of length* L*, forming *K* = *N* $-$ *L* + *1* vectors of length* L*, as shown in Eq. (1):

1 $${X}_{i}={({x}_{i},\dots ,{x}_{i+L-1})}^{T} \left(1\le i\le K\right).$$

These vectors form the trajectory matrix, as shown in Eq. (2):

2 $$X=\left[{X}_{1}:...:{X}_{K}\right]={{(x}_{ij})}_{i,j=1}^{L,K}=\left(\begin{array}{ccc}{x}_{1}& ...& {x}_{K}\\ \vdots & \ddots & \vdots \\ {x}_{L}& \cdots & {x}_{N}\end{array}\right).$$

#### SVD

$L$ Eigenvalues can be obtained by SVD of the above matrix $X{X}^{T}$, and ${\lambda }_{1}\ge {\lambda }_{2}\ge ...\ge {\lambda }_{L}\ge$

$0$ can be obtained in descending order. ${U}_{i}$ is the feature vector. The eigenvector corresponding to the largest eigenvalue is the change trend of the sequence, while the eigenvector corresponding to the smaller eigenvalue is noise. The singular spectrum of the sequence is $\sqrt{{\lambda }_{1}}\ge \sqrt{{\lambda }_{2}}\ge ...\ge \sqrt{{\lambda }_{L}}\ge 0$. Assuming $d=min\{L,K\},{V}_{i}={X}^{T}{U}_{i}/\sqrt{{\lambda }_{i}}$, the elementary matrix ${X}_{i}=\sqrt{{\lambda }_{i}}{U}_{i}{{V}_{i}}^{T}$, the original sequence decomposition is shown in Eq. (3):

3 $$X={X}_{1}+{X}_{2}+...+{X}_{d}.$$

#### Group

Divide the subscript interval $\{\mathrm{1,2},...,d\}$ of the elementary matrix ${X}_{i}$ into *q* disconnected subsets ${I}_{2},...,{I}_{q}$, where $I=\{{i}_{1},{i}_{2},...,{i}_{m}\}$. The synthetic matrix ${X}_{I}={X}_{i1}+{X}_{i2}+...+{X}_{im}$ and each composition matrix of ${I}_{1},{I}_{2},...,{I}_{q}$ are then calculated. Therefore, the decomposition of Eq. (3) is shown in Eq. (4):

4 $$X={X}_{I1}+{X}_{I2}+...+{X}_{Iq}.$$

The selection process of ${I}_{1},{I}_{2},...,{I}_{q}$ is grouping.

#### Recombination

Each matrix ${X}_{Ij}$ obtained by Eq. (4) is converted into a new sequence of length *N*, i.e. the decomposed sequence is obtained. Let $Y$ be a matrix of $L*K$. Let $Y={{(y}_{ij})}_{L*K}$, ${L}^{*}=min\{L,K\}{,K}^{*}=max\{L,K\}$ and $N=K+L-1.\mathrm{ If} L < K,{y}_{ij}^{*}= {y}_{ij}$; otherwise,${y}_{ij}^{*}= {y}_{ji}$. Then, the diagonal averaging formula is used to change $Y$ to time series ${y}_{1},{y}_{2},...,{y}_{N}$. The diagonal averaging formula is shown in Eq. (5):

5 $${y}_{k}=\left\{\begin{array}{c}\frac{1}{k}\sum_{m=1}^{k}{y}_{m,k-m+1}^{*},\,for\,\, 1\le k<{L}^{*},\\ \frac{1}{{L}^{*}}\sum_{m=1}^{{L}^{*}}{y}_{m,k-m+1}^{*},\,for\,\, {L}^{*}\le k<{K}^{*}\\ \frac{1}{N-k+1}\sum_{m=k-{K}^{*}+1}^{N-{K}^{*}+1}{y}_{m,k-m+1}^{*},\,for\,\, {K}^{*}<k\le N\end{array}.\right.$$

Thus, the two-dimensional matrix is transformed into a one-dimensional matrix.

### BiLSTM

**Figure 1:**
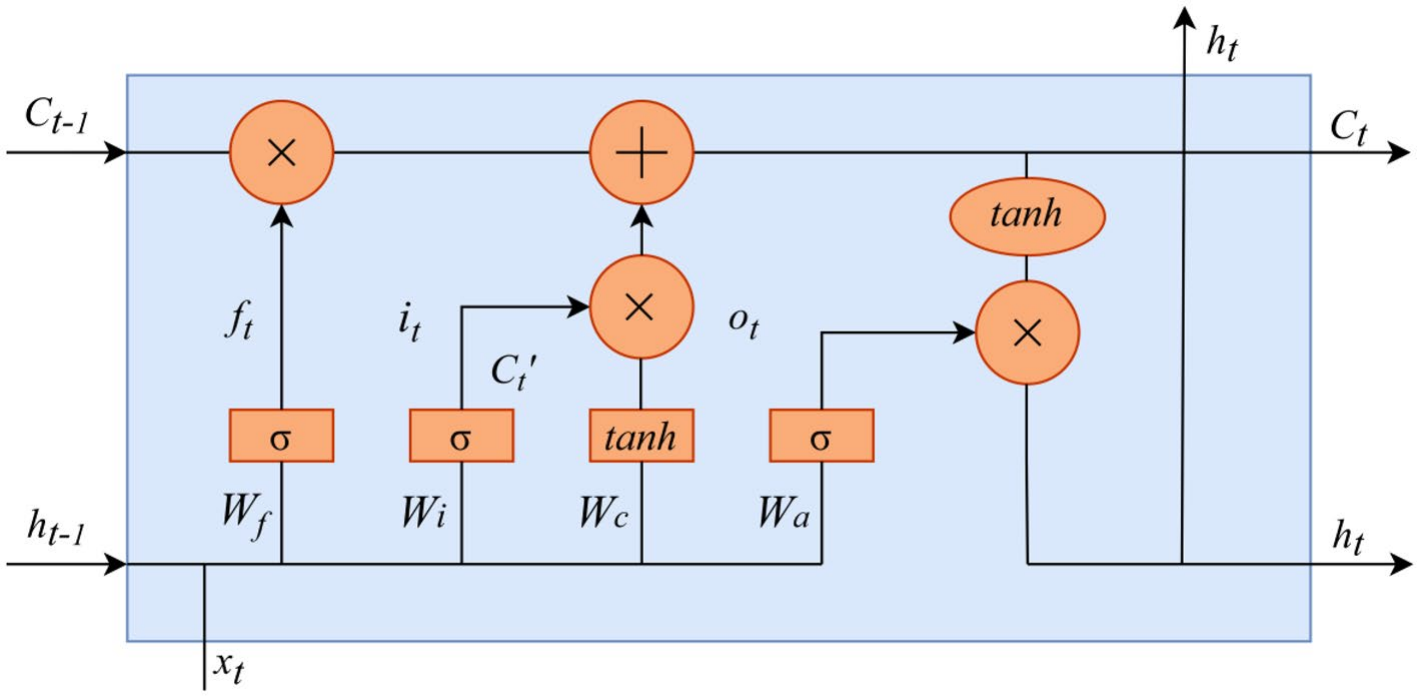
LSTM model structure.

**Figure 2:**
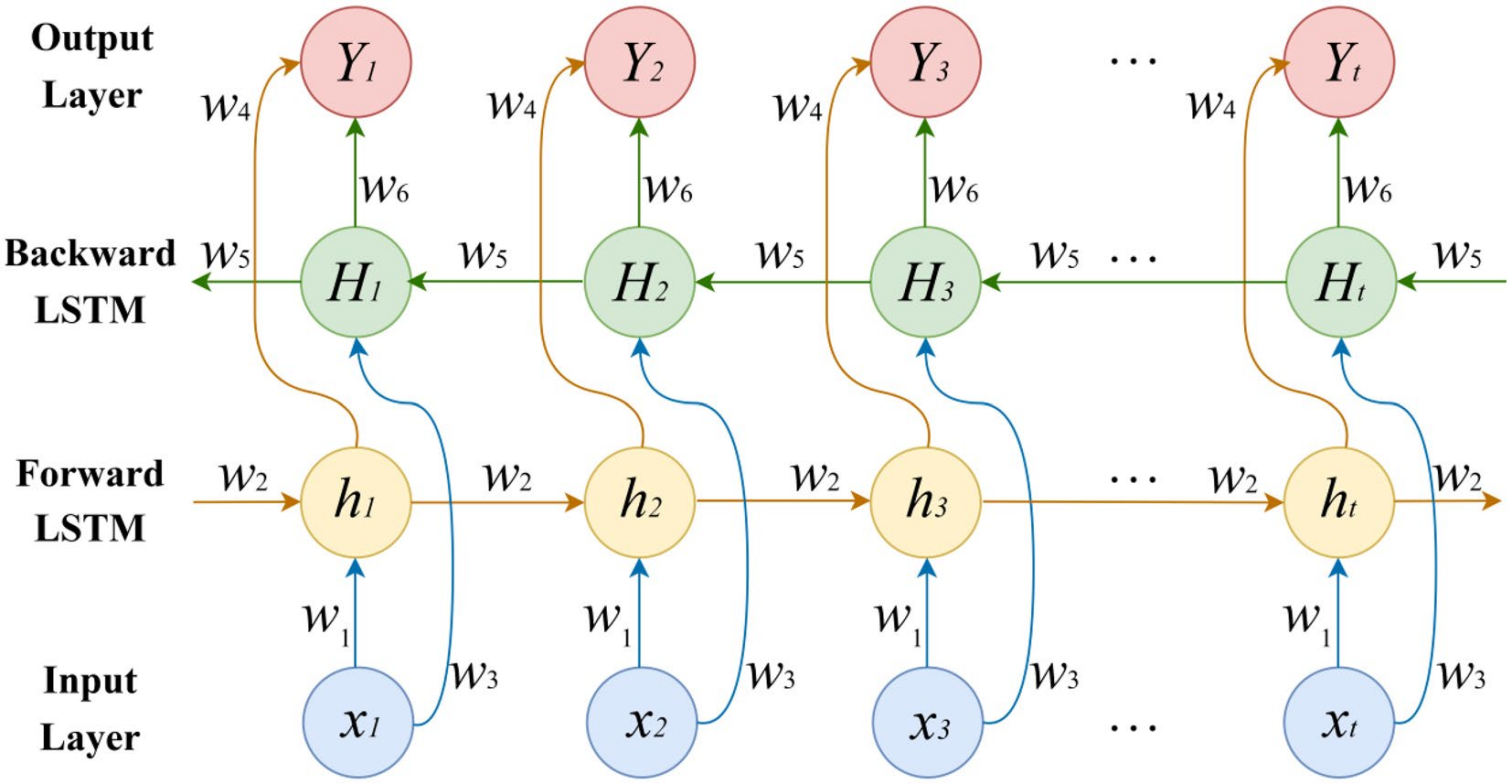
BiLSTM model structure.

BiLSTM^20^ is improved from traditional one-way LSTM optimization. Hochreiter S et al.^21^ established an LSTM model to effectively solve the gradient explosion or disappearance problem of traditional recurrent neural networks. The control mechanism realizes the selective transmission of information; that is, the forget gate, input gate, and output gate are introduced on the basis of traditional RNN so that the model can maintain a more stable error during backpropagation and can continue learning at multiple time steps, thereby improving the accuracy of time series forecasting. Its structure is shown in Fig. 1:

The overall framework of the LSTM model is composed of the input word ${x}_{t}$, cell state ${C}_{t}$, temporary cell state ${{C}_{t}}^{^{\prime}}$, hidden layer state ${h}_{t}$, forgetting gate ${f}_{t}$, memory gate ${i}_{t}$, and output gate ${o}_{t}$ at moment *t*. The calculation process of LSTM can be summarized as follows: by forgetting the information in the cell state and memorizing new information, the information useful for subsequent calculations is transmitted, and the useless information is discarded, and the hidden layer state is output at each time step ${h}_{t}$, where forgetting, memory and output are controlled by the forget gate ${f}_{t}$, memory gate ${i}_{t}$, and output gate ${o}_{t}$ calculated from the hidden layer state ${h}_{t-1}$ at the last moment and the current input ${x}_{t}$. BiLSTM is composed of a forward LSTM layer and a backward LSTM layer. Both will affect the output, which is beneficial to the input of both forward sequence information and backward sequence information. It fully considers past and future information, which is conducive to further improving the accuracy of model predictions. The structure of BiLSTM is shown in Fig. 2:

Where ${x}_{i}(i=1, 2, ...,t)$ represents the input data at the corresponding time, ${h}_{i}(i=1, 2,...,t)$ represents the LSTM hidden state of the forward iteration at the corresponding time, and ${H}_{i}(i=1, 2, ...,t)$ represents the LSTM hidden state of the backward iteration. ${Y}_{i}(i=1, 2,...,t)$ represents the corresponding output data, and ${w}_{i}(i=1, 2,...,6)$ represents the corresponding weight of each layer. The calculation formulas of the final output process of BiLSTM are shown in Eqs. (6), (7), (8):

6 $${h}_{i}={f}_{1}\left({w}_{1}{x}_{i}+{w}_{2}{h}_{i-1}\right),$$

7 $${H}_{i}={f}_{2}\left({w}_{3}{x}_{i}+{w}_{5}{H}_{i+1}\right),$$

8 $${Y}_{i}={f}_{3}\left({w}_{4}{h}_{i}+{w}_{6}{H}_{i}\right),$$

where ${f}_{1}$, ${f}_{2}$ and ${f}_{3}$ are the activation functions corresponding to different layers.

### LightGBM

LightGBM^22^ is an ensemble learning algorithm based on boosting. Compared with other boosting algorithms, it can greatly reduce the training time and memory occupation without reducing the prediction accuracy.

LightGBM mainly uses gradient one-sided sampling (GOSS) and mutually exclusive feature bundling (EFB) to improve the training speed. GOSS selects data with larger gradients from the samples to improve the contribution to computing information gain. EFB combines some features of the data to reduce the data dimension. At the same time, LightGBM mainly uses the histogram algorithm and the leaf growth strategy with a depth limit to reduce memory consumption. The histogram algorithm constructs a histogram with a width of *L* by discretizing continuous floating-point features into *L* integers. When traversing the data, statistics are accumulated in the histogram according to the discretized value as an index, and then the optimal split point is found from the discrete values of the histogram. The leaf growth strategy with depth limit refers to finding a leaf with the largest splitting gain from all the current leaves each time to split and setting a maximum depth limit. While ensuring high efficiency, it prevents model overfitting.

### SSA -BiLSTM-LightGBM

**Figure 3:**
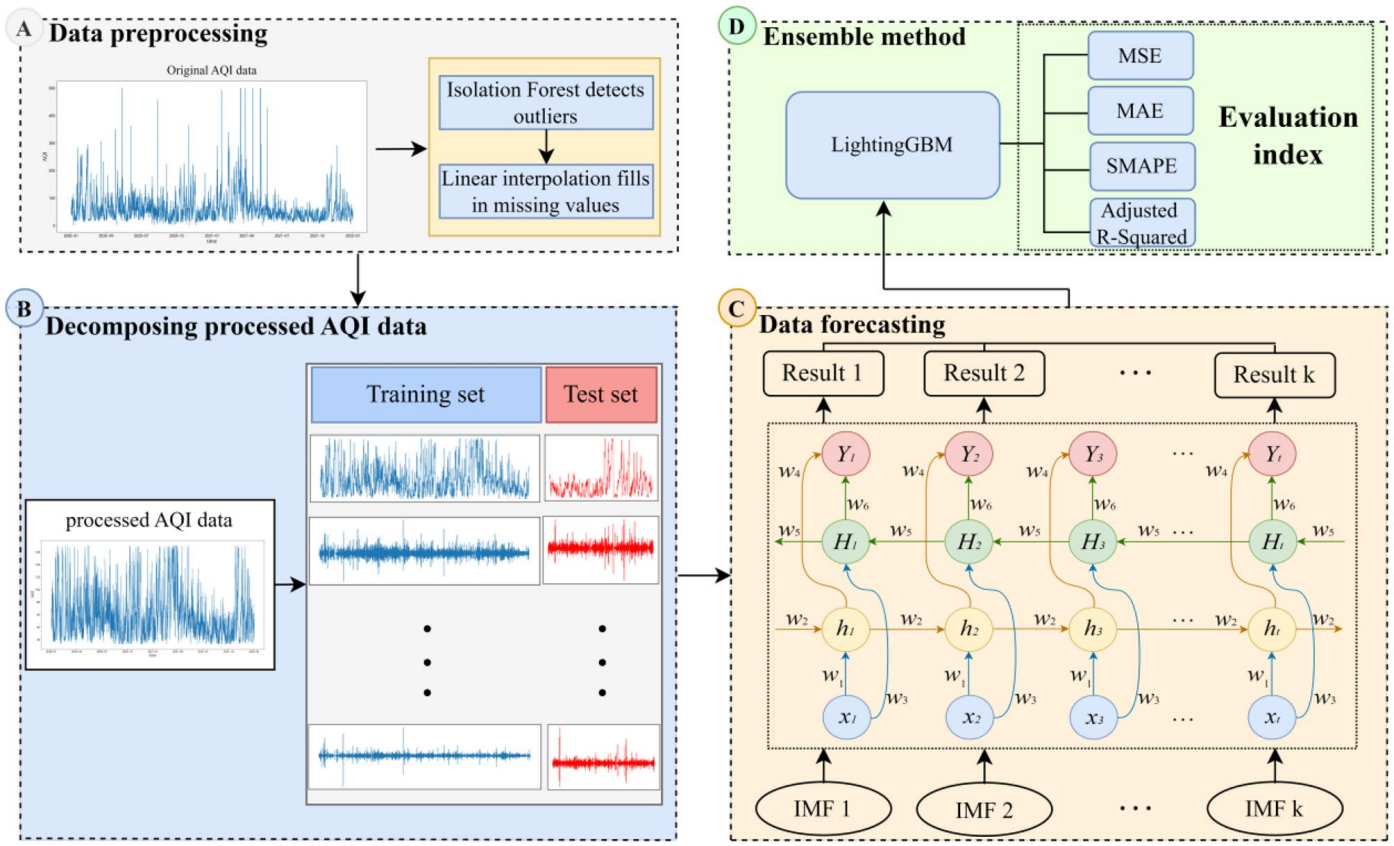
SSA-BiLSTM-LightGBM model structure.

SSA-BiLSTM-LightGBM mainly decomposes trends, oscillation components and noise from a time series based on SVD through SSA to reduce the influence of data instability on the prediction effect and then uses BiLSTM to predict the AQI. The model prediction results are finally integrated through the LightGBM model. The model structure is shown in Fig. 3:

## Experiments

### Data sources

The experimental data used in this paper come from the Beijing AQI data set obtained by monitoring (https://www.aqistudy.cn/), which includes the real AQI of Beijing every hour from 0:00 on January 1, 2020, to 23:00 on December 31, 2021. A total of 17,544 sample points are included. The test task of this experiment is to predict the AQI data value at *t* + *1* h according to all the information of the previous *t* h and use the first 80% of the time series data to train the prediction model. The remaining 20% of the data will be used to verify and test the performance of the prediction model. That is, the training set selected in this paper is the Beijing real-time AQI data from 0:00 on January 1, 2020, to 19:00 on August 7, 2021, and the test set is Beijing real-time AQI data from 20:00 on August 7, 2021, to 23:00 on December 31, 2021.

### Evaluation indicators

To compare the classification performance of different models from multiple levels, four evaluation indicators (RMSE, MAE, SMAPE and adjusted R^2^) are used in this paper. If the RMSE, MAE, and SMAPE of a model are smaller and the adjusted R^2^ is larger, then the prediction effect of the model is better.

The calculation formula of RMSE is shown in Eq. (9):

9 $$\mathrm{RMSE}=\sqrt{\frac{1}{n}\sum_{i=1}^{n}{(\widehat{{y}_{i}}-{y}_{i})}^{2}.}$$

The calculation formula of MAE is shown in Eq. (10):

10 $$\mathrm{MAE}=\frac{1}{n}\sum_{i=1}^{n}\left|\widehat{{y}_{i}}-{y}_{i}\right|.$$

The calculation formula of SMAPE is shown in Eq. (11):

11 $$\mathrm{SMAPE}=\frac{100\%}{n}\sum_{i=1}^{n}\frac{\left|\widehat{{y}_{i}}-{y}_{i}\right|}{(\left|\widehat{{y}_{i}}\right|+\left|{y}_{i}\right|)/2}.$$

The calculation formula of adjusted R^2^ is shown in Eq. (12):

12 $$\mathrm{adjusted }\,\,{\mathrm{R}}^{2}=1-\frac{\sum_{i=1}^{n}{{\left({y}_{i}-\widehat{{y}_{i}}\right)}^{2}}/{(n-k-1)}}{{\sum_{i=1}^{n}{\left({y}_{i}-\overline{y}\right)}^{2}}/{(n-1)}}.$$

### Data preprocessing

#### Outlier detection

**Figure 4:**
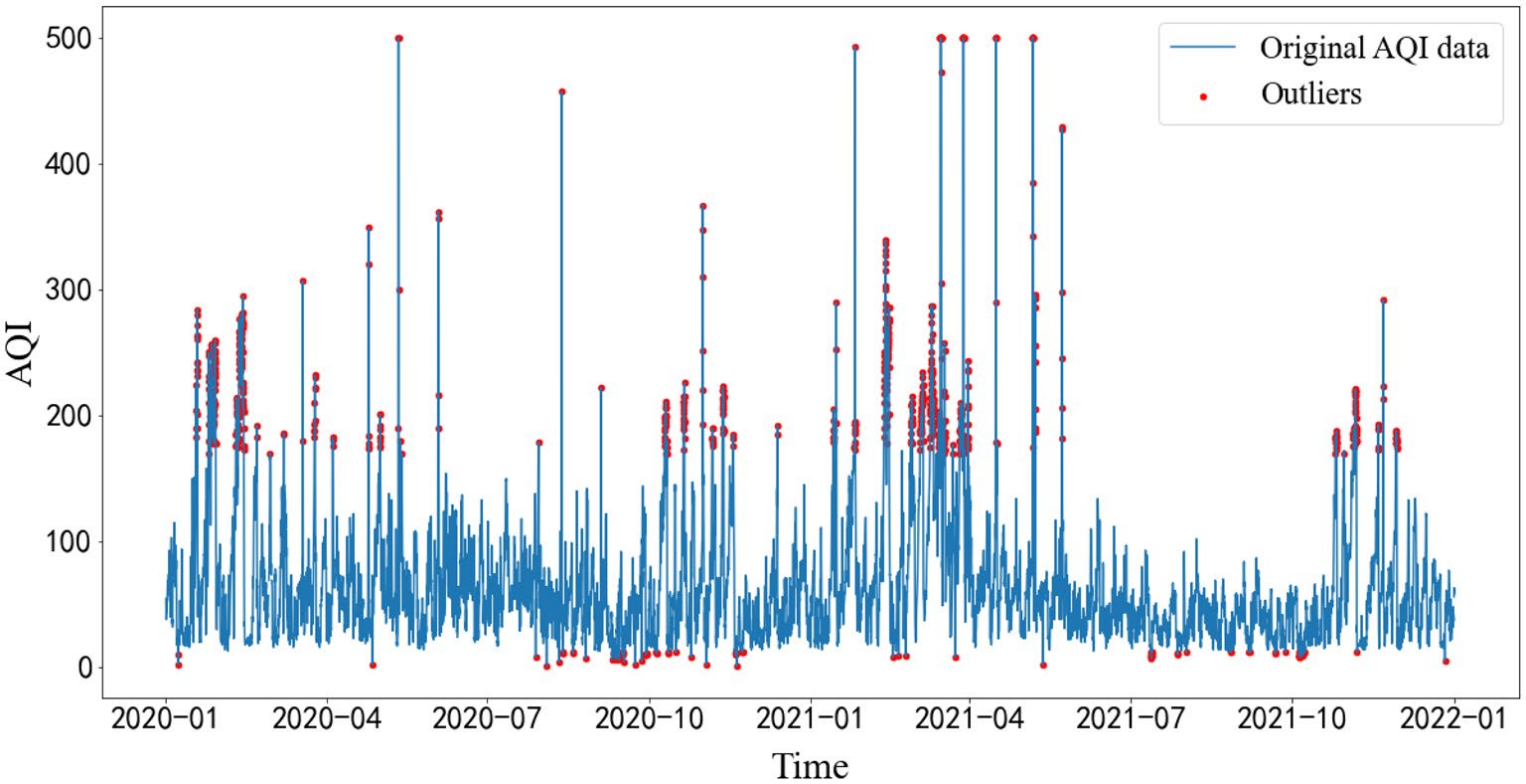
Outlier detection.

Outliers in time series analysis have a large negative impact on time series forecasting. In the training process, the detection and processing of outliers is very important. The isolation forest algorithm is an anomaly detection method based on Ensemble. It is an unsupervised learning algorithm with linear time complexity, high accuracy and fast speed in processing big data. Compared with other commonly used outlier detection algorithms, such as statistical methods, classification-based methods, and clustering-based methods, these traditional algorithms usually build a model for normal data and then consider data that do not conform to this model as outliers. However, isolation forest (iForest) can explicitly find abnormal data without building a model for normal data. This reduces memory and increases operation speed. Therefore, this paper chooses the isolation forest algorithm for outlier detection. The test results are shown in Fig. 4:

#### Outlier handling

In the outlier handling part, this paper uses linear interpolation to fill the identified outliers by removing them and treating them as missing values. Compared with other interpolation methods, the forward fill interpolation method does not consider the fact that the actual AQI data change with time, while other polynomial interpolation, spline interpolation, etc. are found to generate more outliers after application to these data. Therefore, this paper chooses linear interpolation as the filling method.

#### Data normalization

After the data are normalized, the convergence speed of the loss function can be improved, gradient explosion can be prevented, and the calculation accuracy can be improved. In this paper, min–max normalization is used to normalize the data to [0, 1] before data prediction. Then, the normalized data are input into the prediction model to obtain the normalized prediction results and normalized to obtain the actual prediction results.

### Single model prediction

**Figure 5:**
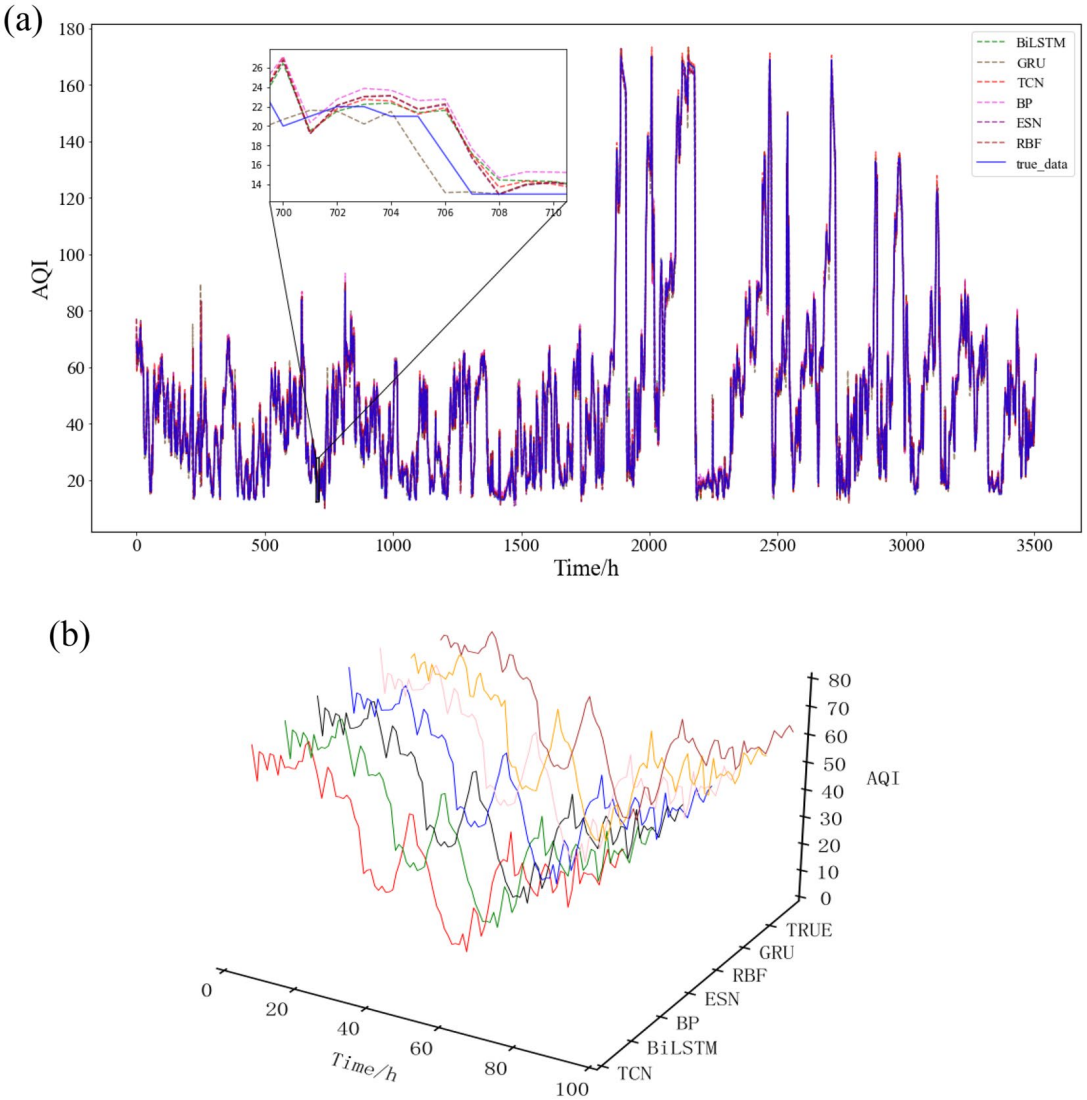
Prediction experimental results of a single model: (**a**) 2d plot; (**b**) 3d plot.

**Figure 6:**
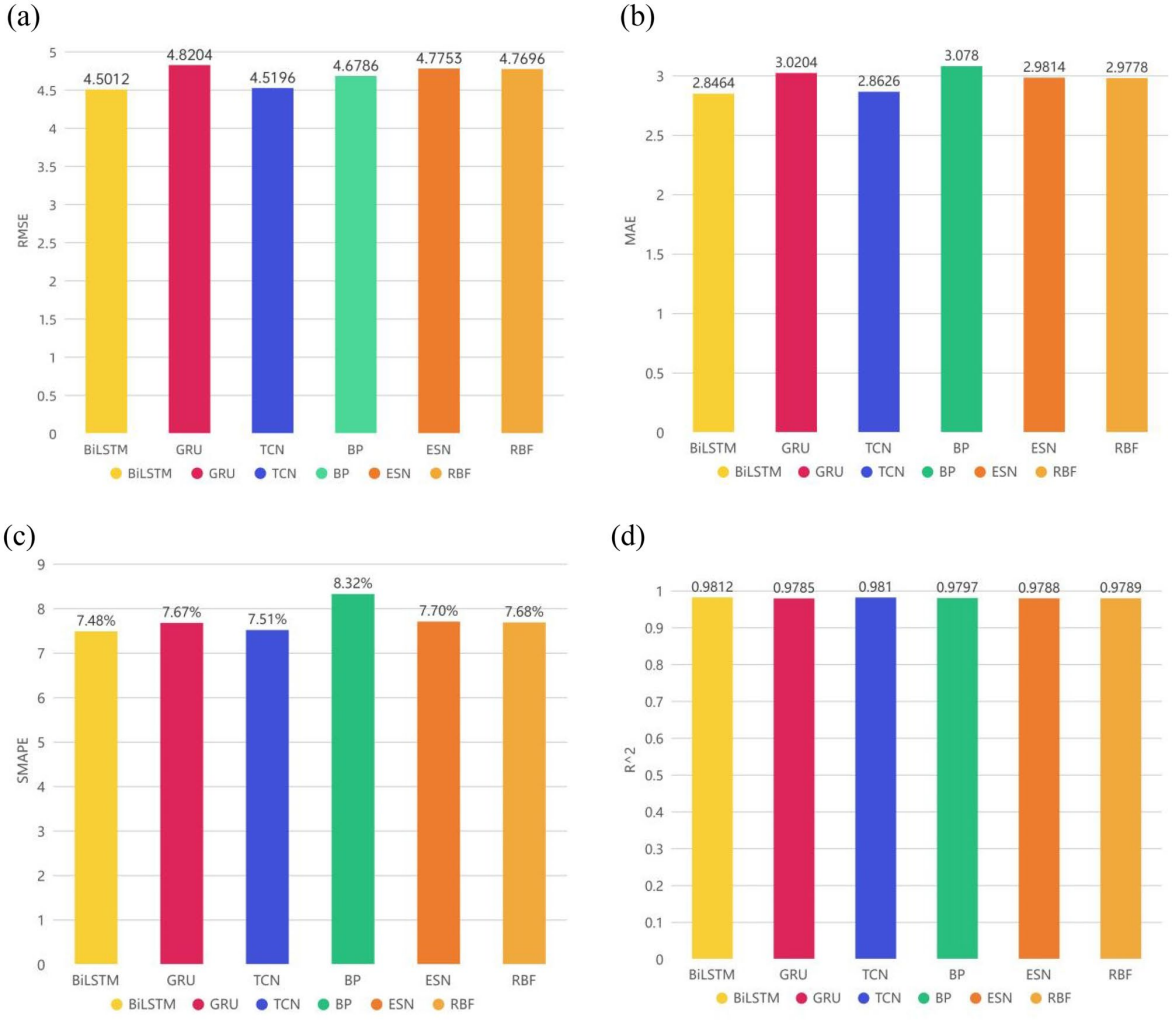
Column chart of single model prediction evaluation index comparison: (**a**) RMSE; (**b**) MAE; (**c**) SMAPE; (**d**) adjusted R^2^.

To compare the prediction performance of different models on AQI data, six different traditional neural network prediction models (BiLSTM, GRU, TCN, BP, ESN and RBF) were selected for experiments. The experimental results of each model on the test set (Beijing real-time AQI data from 20:00 on August 7, 2021, to 23:00 on December 31, 2021) are shown in Fig. 5:

The evaluation results of the six prediction models are shown in Table 1 and Fig. 6.

**Table 1:** Evaluation results of single prediction model indicators.

Models	RMSE	MAE	SMAPE	$\mathrm{Adjusted }\,\,{\mathrm{R}}^{2}$
**BiLSTM**	**4.5012**	**2.8464**	**7.4806%**	**0.9812**
GRU	4.8204	3.0204	7.6671%	0.9785
TCN	4.5196	2.8626	7.5057%	0.9810
BP	4.6786	3.0780	8.3241%	0.9797
ESN	4.7753	2.9814	7.7000%	0.9788
RBF	4.7696	2.9778	7.6751%	0.9789

The significant values are in bold.

After analysis, it is found that the evaluation results of each evaluation index of BiLSTM and the temporal convolutional network (TCN) are optimal compared with the index evaluation results of other models. This shows that the two deep neural network models, BiLSTM and TCN, have excellent performance in predicting the time series data of the AQI. The possible reasons are as follows: (1) For problem tasks that are sensitive to time series, RNN (such as LSTM) may usually be more suitable than back propagation (BP) and radial basis function (RBF); at the same time, LSTM has several more gates than RNN, and even one more gate than gated recurrent unit (GRU), which can be used to control the flow of information, and thus may improve the prediction performance; echo state network (ESN) ended up showing the worst prediction performance of the six models, probably because ESN is not suitable for this data set. (2) BiLSTM adds a reverse operation based on LSTM, which can better capture the relationship between time series features than LSTM. It not only considers the previous information but also takes the latter information into consideration. For time series, the prediction may sometimes need to be determined by several previous inputs and several subsequent inputs, which will make the prediction more accurate. (3) First, TCN can perform parallel processing, while LSTM can only perform sequential processing. Second, the TCN has a flexible receptive field, which is determined by the number of layers, the size of the convolution kernel, and the expansion coefficient. It can be flexibly customized according to the different characteristics of different tasks. Then, the TCN does not have the problem of vanishing gradients and exploding gradients as often as LSTM. Some research results show that the TCN after introducing architectural elements such as atrous convolution and residual connections is more effective than recursive architectures such as LSTM in different time series modeling tasks.

Therefore, according to the above analysis, BiLSTM and TCN are improved in different aspects compared with LSTM, and according to existing experiments, the AQI is mostly nonstationary, so this article will combine BiLSTM and TCN, citing decomposition and ensemble algorithms to make further predictions on this data set.

### Hybrid model prediction

#### SSA

**Figure 7:**
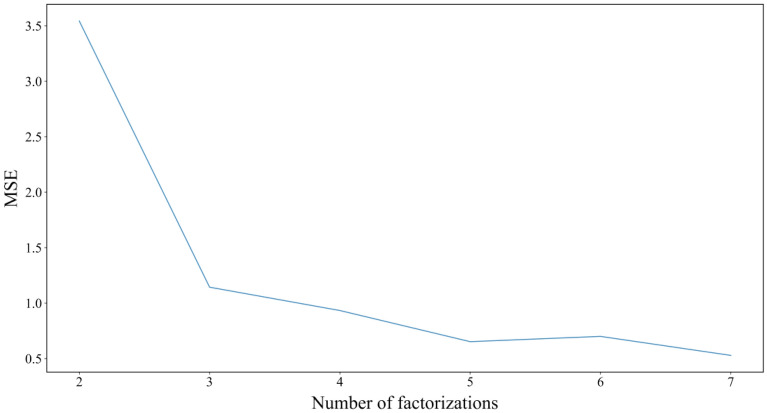
Index evaluation of different decomposition numbers in the BiLSTM training set.

**Figure 8:**
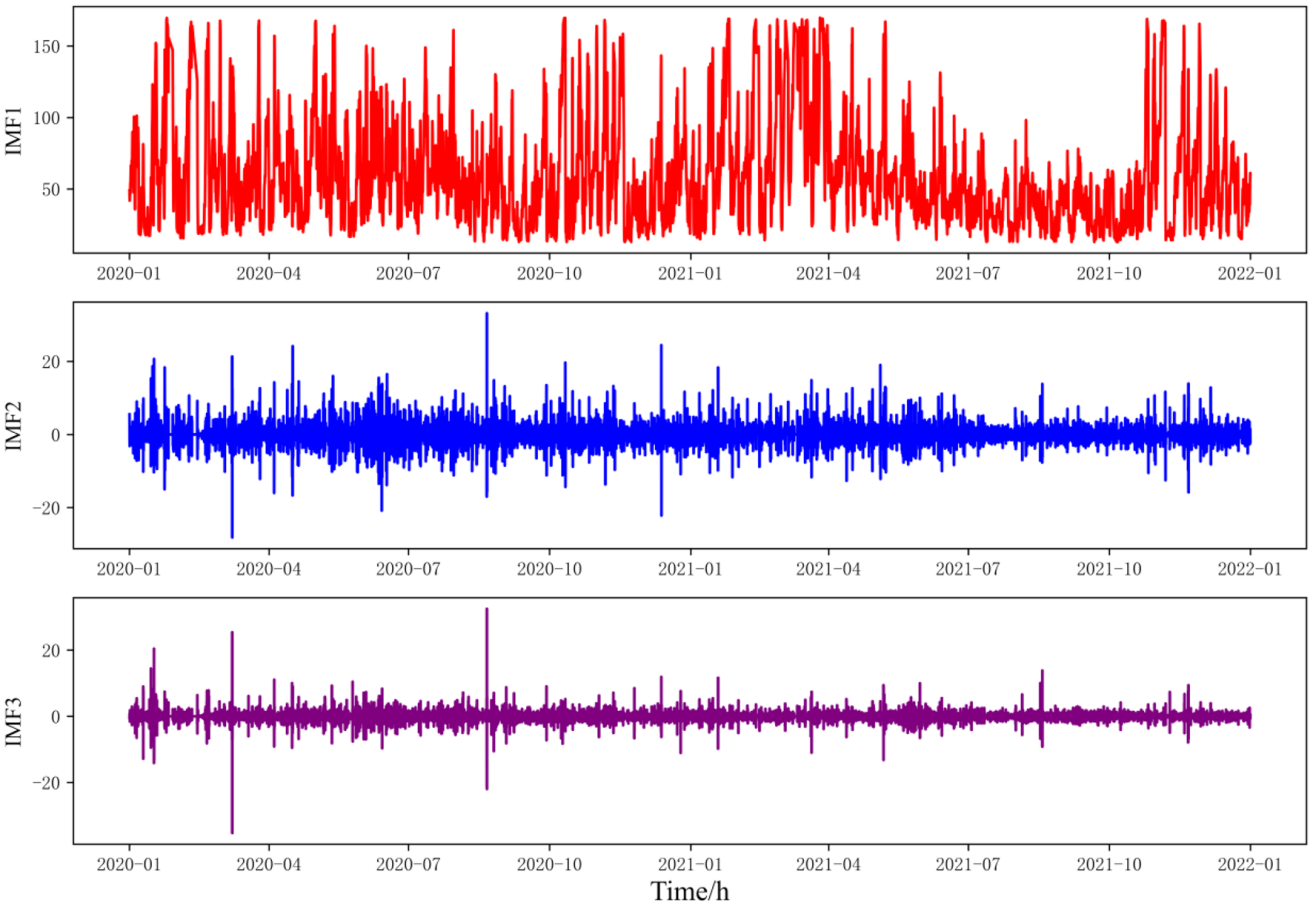
SSA decomposition results.

Regarding the setting of the number of SSA decompositions, this paper decomposes the SSA into sequences of different numbers, uses BiLSTM to predict them, and then limits the number of SSA decompositions according to the prediction effect in the training set. The RMSE of different numbers of sequences formed by SSA decomposition in the training set is shown in Fig. 7:

It can be seen from the figure that after the original sequence is decomposed into multiple. The RMSE of IMF components in the training set decreases with the increase in the number of decompositions. However, it has an obvious inflection point when the number of decompositions is 3, and the change is relatively stable thereafter. Considering the risk of overfitting, this paper chooses the number of decompositions as 3 as the number of SSA decompositions and conducts the following experiments. Its decomposition sequence is shown in Fig. 8:

#### Comparative analysis of combination models

To verify the superiority of the prediction performance of the SSA-BiLSTM-LightGBM model proposed in this paper on this data set. In this paper, three decomposition models, two prediction models and two integrated regression models are selected to combine different strategies. After the combination models are formed, they are compared with the target model proposed in this paper, and the evaluation indicators of each model are shown in Table 2:

**Table 2:** Prediction results of the combined model.

Models	RMSE	MAE	SMAPE	Adjusted R^2^
SSA-BiLSTM	0.9814	0.7312	1.7491%	0.9990
CEEMDAN-BiLSTM	4.1013	3.0204	7.5828%	0.9843
EMD-BiLSTM	4.4190	3.0033	7.9112%	0.9818
CEEMDAN-TCN	5.5501	4.4608	11.1205%	0.9712
EMD-TCN	5.3752	4.0268	10.4293%	0.9730
SSA-TCN	1.3141	0.9134	2.6371%	0.9982
SSA-BiLSTM-XGBoost	0.7446	0.4732	1.2958%	0.9993
**SSA-BiLSTM-LightGBM**	**0.6897**	**0.4718**	**1.2712%**	**0.9995**
SSA-BiLSTM-AdaBoost	0.8807	0.4303	1.1453%	0.9991
SSA-TCN-AdaBoost	1.2036	0.7038	1.9766%	0.9985
SSA-TCN-XGBoost	1.0258	0.6855	1.9427%	0.9989
SSA-TCN-LightGBM	0.9853	0.6789	1.9018%	0.9990

The significant values are in bold.

**Figure 9:**
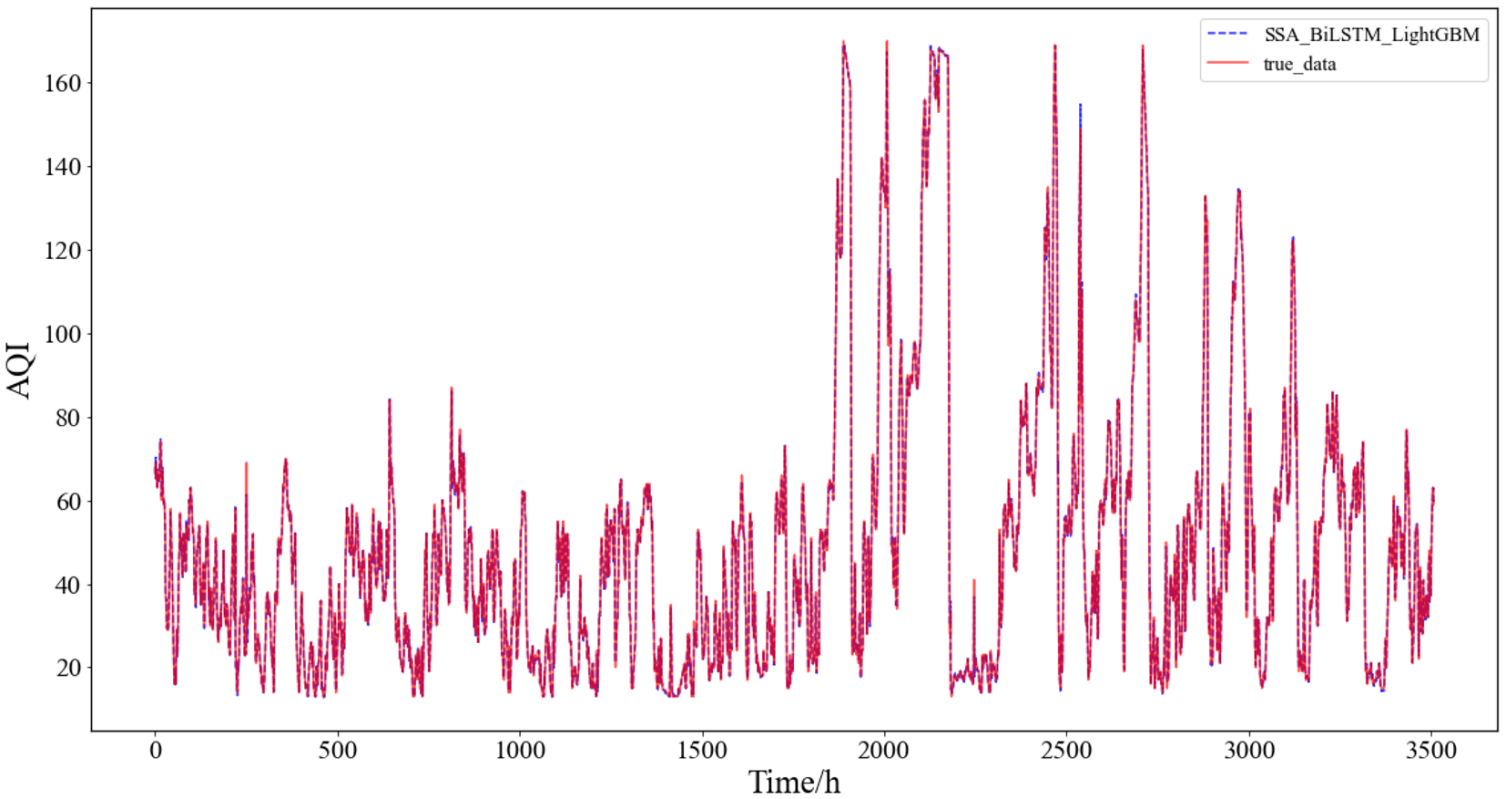
SSA-BiLSTM-LightGBM prediction result.

**Figure 10:**
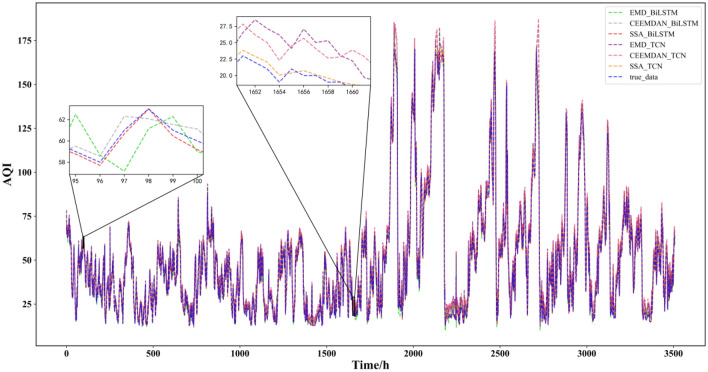
Prediction experimental results of combination models based on decomposition.

**Figure 11:**
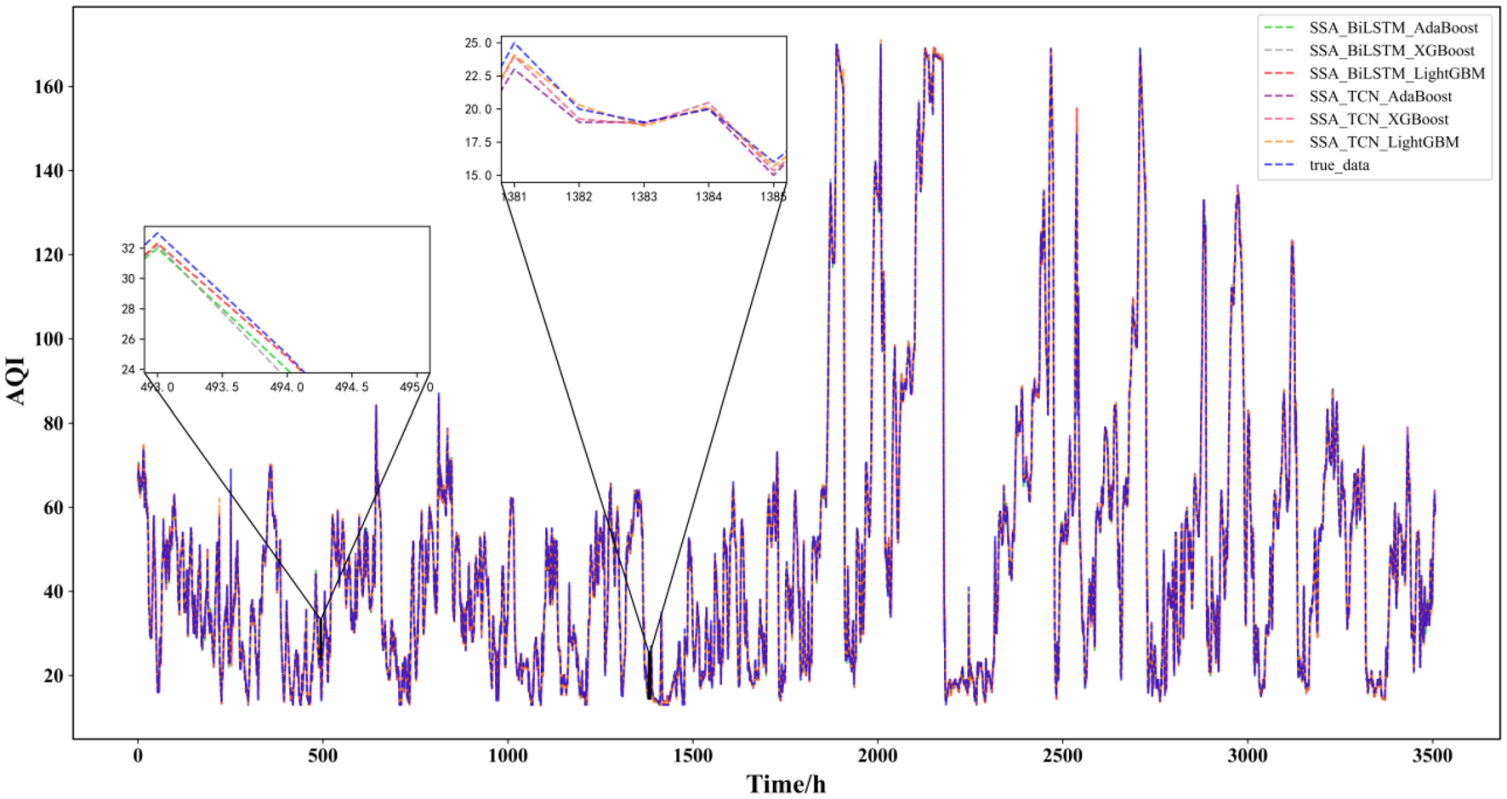
Prediction experimental results of combination models based on integration.

**Figure 12:**
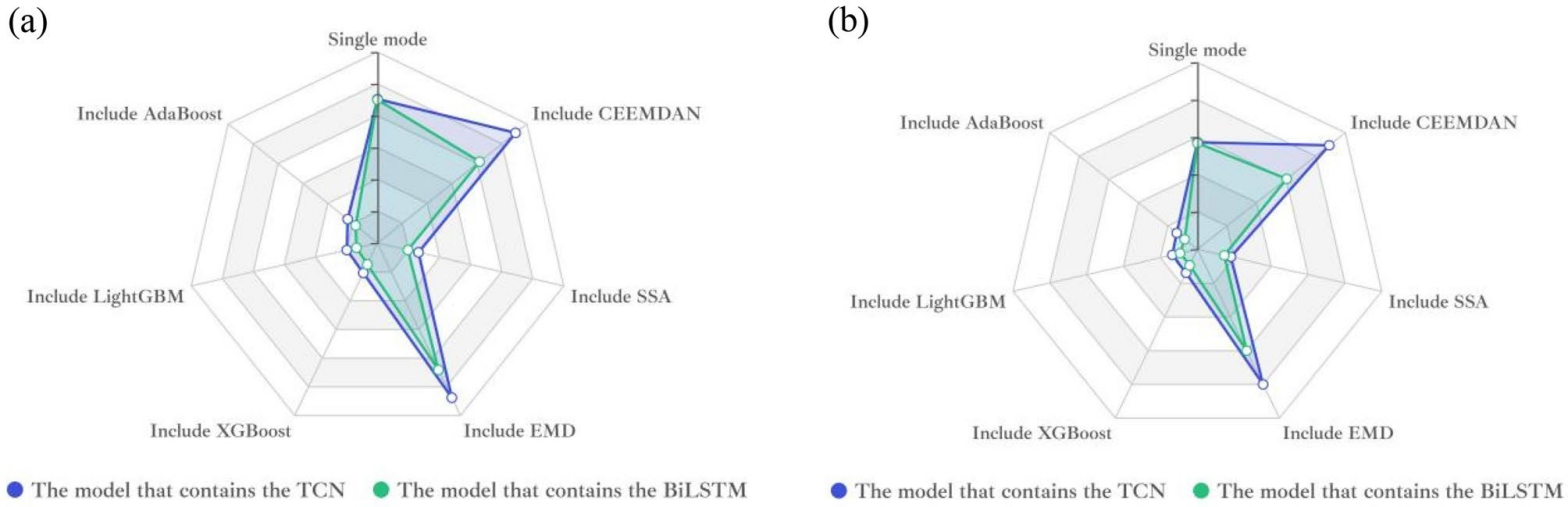
The comparison results of model’s evaluation indicators: (**a**) RMSE; (**b**) MAE.

**Figure 13:**
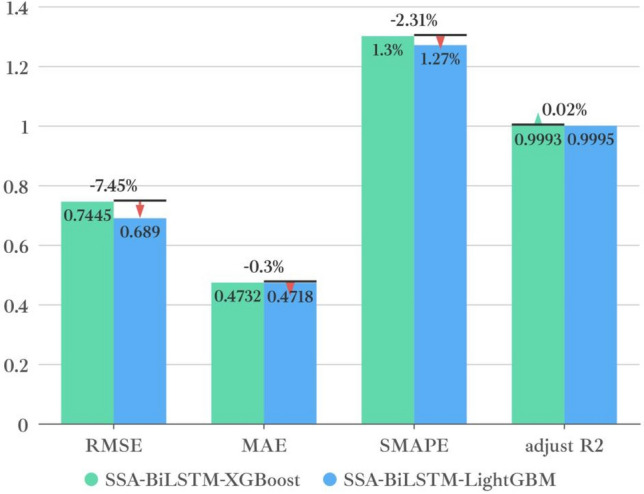
The comparison results of the evaluation indicators.

From the table, it can be concluded that SSA-BiLSTM-LightGBM has the best prediction effect compared with other comparison models, and its prediction results are shown in Fig. 9:

This paper reflects the superiority of the SSA-BiLSTM-LightGBM model from four aspects based on the comparative model. According to the table, the results can be analyzed and concluded as follows:

- (1) First, the combined model containing SSA was compared with other models, and it was found that the prediction performance of the decomposition model containing SSA was the best. This shows that for this data set, SSA is more suitable than empirical mode decomposition (EMD) and CEEMDAN in decomposing series before forecasting. This may be because SSA is model-free, and when it is applied, it does not require stability for the time series, nor does it assume a parametric model, so it can be widely used in various time series. Their prediction results are shown in Fig. 10:
- (2) Second, after comparing and analyzing LightGBM and other models, it is found that the prediction performance of the SSA-BiLSTM-XGBoost model is the best, which fully reflects that compared with models such as adaptive boosting (AdaBoost) and extreme gradient boosting (XGBoost), LightGBM has a better performance in the process of integrating results. The possible reason may be that LightGBM adopts a leafwise growth strategy, and each time it finds a leaf split with the largest split gain from all the current leaves. Therefore, it can reduce more errors and obtain better accuracy. At the same time, a maximum depth limit is added on top of leafwise to prevent overfitting while ensuring high efficiency. Their prediction results are shown in Fig. 11:
- (3) Then, all the combined models containing BiLSTM and all the combined models containing TCN were compared, and it was found that although the two had little difference in the prediction effect, the combined models containing BiLSTM for each strategy were slightly better than the combined models containing TCN, which shows that BiLSTM is more suitable for prediction than TCN for the AQI data set. This may be because although the TCN can view historical information and future information through inflation, BiLSTM may be more adequate and appropriate to utilize future and past information for this data set. The comparison results of their evaluation indicators are shown in Fig. 12:
- (4) Finally, comparing the SSA-BiLSTM-LightGBM model with SSA-BiLSTM-XGBoost, which ranks second in prediction performance, it can be found that the RMSE of the SSA-BiLSTM-LightGBM model is reduced by 7.37% compared with the SSA-BiLSTM-XGBoost model, and the MAE is reduced by 0.29%. SMAPE is reduced by 1.90%, which shows that the model has a significant improvement in model evaluation indicators compared with other comparison models, indicating that the model has higher prediction accuracy and better prediction performance than other comparison models. The comparison results of their evaluation indicators are shown in Fig. 13:

## Multistep predictive analysis

If the model proposed in this paper can have a good effect on multistep prediction, then the model will be better applied in real-life processes. Therefore, this paper explores the prediction effect of this model in multistep prediction. In this multistep prediction experiment, the Beijing AQI data set obtained by monitoring is still selected as the experimental data, which includes the real hourly AQI of Beijing from 0:00 on January 1, 2020, to 23:00 on December 31, 2021. The data contain a total of 17,544 sample points. This experiment uses the first 80% of the time series data to train the prediction model. The remaining 20% of the data will be used to verify and test the performance of the prediction model. The final prediction results on the test set are shown in Table 3.

**Table 3:** The evaluation results of SSA-BiLSTM-LightGBM multistep prediction.

Step size	RMSE	MAE	SMAPE	Adjusted R^2^
1 h	0.6897	0.4718	1.2712%	0.9995
2 h	2.6772	2.2163	6.1351%	0.9938
3 h	6.1321	4.3600	10.8971%	0.9659

From Table 3, it is clear that although the accuracy of the SSA-BiLSTM-LightGBM model proposed in this paper decreases somewhat compared with the single-step prediction, it still has a good prediction effect in the multistep prediction, indicating that the model has certain effectiveness and reference significance in the single-step prediction and multistep prediction.

## Discussion and conclusion

Accurate AQI data prediction is of great significance to atmospheric management and residents' health. The traditional time series prediction model also has large prediction errors in use that increasingly fails to meet the needs of current production and life. However, the neural network represented by LSTM shows excellent prediction performance in time series prediction. Meanwhile, decomposition algorithms represented by EMD have been proved by many studies to play a significant positive role in improving the accuracy of AQI prediction. On the basis of previous studies, an integrated model based on EMD decomposition and BiLSTM prediction is proposed in this paper. We use SSA to decompose the sequence, input the decomposed sequence into BiLSTM for prediction, and then use LightGBM to integrate the prediction results to further improve the prediction accuracy. We use the detected AQI data of Beijing every hour from 2019 to 2020 to construct and analyze all models, and decide to use the data of the first 24 h to make one-step prediction of AQI, and extend its application to multistep prediction. The experimental results show that the prediction effect of SSA-BiLSTM-LightGBM on the test set is good. After analysis, RMSE, MAE and SMAPE of SSA-BiLSTM-LightGBM are reduced by 84.67%, 83.42% and 83.02% compared with the basic model BiLSTM. Compared with SSA-BiLSTM that directly added the decomposed prediction models, RMSE, MAE and SMAPE of SSA-BiLSTM-LightGBM are reduced by 29.72%, 35.48% and 27.43%. We think that the reason why this model can achieve such good prediction performance is that SSA can decompose data features more effectively, and LightGBM can integrate forecast data better by combining data features.

In addition, in the case analysis, we can conclude that BiLSTM is more suitable for predicting AQI time series than other neural network prediction models that may be due to the addition of reverse operation that can better capture the relationship between sequential features and thus make the prediction more accurate. When BiLSTM is used for prediction, SSA is firstly used for feature extraction to decompose AQI time series data into decomposed trend, oscillation component and noise sequence data that is conducive to improving prediction accuracy. Moreover, LightGBM adopts leaf-wise growth strategy in the process of integrating BiLSTM results that can reduce more errors and obtain better accuracy. At the same time, it can ensure high efficiency and prevent overfitting. Therefore, the prediction effect of SSA-BiLSTM-LightGBM in this AQI dataset is superior to other corresponding comparison models.

The model proposed in this paper still has the following problems: (1) We only conducted prediction research on the AQI data of Beijing. If the AQI data of other regions or cities can be collected in the future, the good prediction performance and strong generalization ability of SSA-BiLSTM-LightGBM model will be further verified. (2) There are many external factors affecting the air quality (AQI) index, such as various meteorological indicators that are not considered in this paper. In the future, various influencing factors can be introduced into the model to improve the accuracy of the model. In conclusion, this study shows that our proposed model can achieve higher accuracy than the traditional single model such as BiLSTM, and the method based on EMD decomposition and LightGBM integration has better performance than other decomposition integration methods. Moreover, the model construction is not complicated, and it is worthy of application in practice.

## Data Availability

The data sets used and/or analyzed during the current study are available from the corresponding author on reasonable request.
